# Treatment with Imiquimod enhances antitumor immunity induced by therapeutic HPV DNA vaccination

**DOI:** 10.1186/1423-0127-17-32

**Published:** 2010-04-28

**Authors:** Chi-Mu Chuang, Archana Monie, Chien-Fu Hung, T-C Wu

**Affiliations:** 1 Department of Pathology, Johns Hopkins Medical Institutions, Baltimore, Maryland, USA; 2 Department of Obstetrics and Gynecology, Johns Hopkins Medical Institutions, Baltimore, Maryland, USA; 3 Department of Molecular Microbiology and Immunology, Johns Hopkins Medical Institutions, Baltimore, Maryland, USA; 4 Department of Oncology, Johns Hopkins Medical Institutions, Baltimore, Maryland, USA; 5 Department of Obstetrics and Gynecology, Taipei Veterans General Hospital, Taipei, Taiwan; 6 School of Medicine, National Yang-Ming University, Taipei, Taiwan

## Abstract

**Background:**

There is an urgent need to develop new innovative therapies for the control of advanced cancer. The combination of antigen-specific immunotherapy with the employment of immunomodulatory agents has emerged as a potentially plausible approach for the control of advanced cancer.

**Methods:**

In the current study, we explored the combination of the DNA vaccine encoding calreticulin (CRT) linked to human papillomavirus type 16 (HPV-16) E7 antigen (CRT/E7) with the TLR7 agonist imiquimod for their ability to generate E7-specific immune responses and antitumor effects in tumor-bearing mice.

**Results:**

We observed that treatment with CRT/E7 DNA in combination with imiquimod leads to an enhancement in the E7-specific CD8+ T cell immune responses and a decrease in the number of myeloid-derived suppressor cells in the tumor microenvironment of tumor-bearing mice. Furthermore, treatment with CRT/E7 DNA in combination with imiquimod leads to significantly improved antitumor effects and prolonged survival in treated mice. In addition, treatment with imiquimod led to increased number of NK1.1+ cells and F4/80+ cells in the tumor microenvironment. Macrophages and NK1.1+ cells were found to play an important role in the antitumor effects mediated by treatment with CRT/E7 DNA in combination with imiquimod.

**Conclusions:**

Thus, our data suggests that the combination of therapeutic HPV DNA vaccination with topical treatment with the TLR7 agonist imiquimod enhances the antitumor immunity induced by DNA vaccination. The current study has significant implications for future clinical translation.

## Background

Advanced stage cancers are difficult to control using conventional therapies such as chemotherapy, surgery and radiation. Therefore, new innovative therapies are urgently required in order to combat the high mortality and morbidity associated with cancers. Antigen-specific immunotherapy has emerged as an attractive approach for the treatment of cancers since it has the ability to specifically eradicate systemic tumors and control metastases without damaging normal cells. For example, in a recent study, immunotherapy using synthetic long peptide vaccine derived from human papillomavirus type 16 (HPV-16) E6 and E7 antigens has led to significant clinical responses in patients with precancerous lesions of gynecological malignancies [1].

DNA vaccination has become a potentially promising approach for antigen-specific immunotherapy due to its safety, stability and ease of preparation (for review, see [2,3]). We have previously developed several innovative strategies to enhance DNA vaccine potency by directly targeting the DNA into the dendritic cells (DCs) in vivo via gene gun as well as by modifying the properties of antigen-expressing DCs (for review see [4,5]).

One of the strategies to enhance DNA vaccine potency uses intracellular targeting strategies to enhance MHC class I antigen presentation and processing in DCs. Previously, we have studied the linkage of calreticulin (CRT), a Ca^2+^-binding protein located in the endoplasmic reticulum (ER) (for review, see [6]) to several antigens, including human papillomavirus type-16 (HPV-16) E7 [7,8], E6 [9], and nucleocapsid protein of severe acute respiratory syndrome coronavirus [10]. Intradermal administration of DNA encoding CRT linked to any of these target antigens led to a significant increase in the antigen-specific CD8+ T cell immune responses and impressive antitumor effects in vaccinated mice. Thus, CRT has been shown to be highly potent in enhancing the antigen-specific immune responses and antitumor effects generated by DNA vaccination in several preclinical models.

Another important innovative cancer therapy involves the employment of immunomodulatory agents such as imiquimod (for reviews see [11,12]). The exact mechanism of action in which imiquimod and its analogs activate the immune system is still under active investigation. Nevertheless, it is known that imiquimod activates immune cells through the toll-like receptor 7 (TLR7), commonly involved in pathogen recognition, on the cell surface [13,14]. Cells activated by imiquimod via TLR-7 secrete cytokines such as IFN-α, IL-6 and TNF-α [15]. There is evidence that imiquimod, when applied to skin, can lead to the activation of Langerhans cells, which subsequently migrates to local lymph nodes to activate the adaptive immune system [16]. Other cell types activated by imiquimod include NK cells, macrophages and B-lymphocytes [16].

In the current study, we hypothesize that the combination of the DNA vaccine encoding CRT linked to HPV-16 E7 (CRT/E7) with topical application of imiquimod at the site of the tumor will lead to increased infiltration of effectior immune cells at the site of the tumor, resulting in enhanced antitumor effects against E7-expressing tumors in a preclinical model. We observed that treatment with CRT/E7 DNA in combination with imiquimod leads to decrease in the number of myeloid-derived suppressor cells (MDSCs) but not T regulatory cells in the tumor microenvironment of tumor-bearing mice. Treatment with CRT/E7 DNA in combination with imiquimod was also found to lead to an enhancement in the E7-specific CD8+ T cell immune responses in tumor-bearing mice. Furthermore, treatment with CRT/E7 DNA in combination with imiquimod leads to significantly improved antitumor effects and prolonged survival in treated mice. In addition, treatment with imiquimod led to increased number of NK1.1+ cells and F4/80+ cells in the tumor microenvironment. Furthermore, macrophages and NK1.1+ cells were found to play an important role in the antitumor effects mediated by treatment with CRT/E7 DNA in combination with imiquimod. The clinical implications of the current study are discussed.

## Methods

### Mice and cell line

Female C57BL/6 mice (H-2K^b ^and I-A^b^), 5 to 6 wks of age, were purchased from the National Cancer Institute. All of the mice were maintained under specific pathogen-free conditions in the animal facility at Johns Hopkins Hospital or Taipei Veterans General Hospital. Animals were used in compliance with institutional animal health care regulations, and all animal experimental procedures were approved by the Johns Hopkins Institutional Animal Care and Use Committee and Taipei Veterans General Hospital Experimental Animal Center.

The production and maintenance of TC-1 cells have been described previously [17]. TC-1 cells were grown in RPMI 1640, supplemented with 10% (v/v) fetal bovine serum, 50 units/mL penicillin/streptomycin, 2 mmol/L L-glutamine, 1 mmol/L sodium pyruvate, 2 mmol/L nonessential amino acids, and 0.4 mg/mL G418 at 37°C with 5% CO2. The TC-1 cells have been tested and shown to be free of mycoplasma and other contamination.

### Plasmid DNA constructs

The generation of recombinant plasmid pcDNA3-CRT/E7 has been described previously [7]. The accuracy of the DNA construct was confirmed by DNA sequencing. For the gene gun-mediated intradermal vaccination, 2 μg/mouse of recombinant plasmid DNA were delivered to the shaved abdominal region of C57BL/6 mice using a helium-driven gene gun (Bio-Rad) with a previously described protocol [18].

### Flow cytometry analysis

For characterization of regulatory T cells and MDSC in tumor microenvironment, C57BL/6 mice were divided into four groups (5/group: untreated, imiquimod alone, CRT/E7 alone, and combined group). All mice were inoculated with TC-1 cells (5 × 10^4^/mouse) s.c. over right leg at d0. For the untreated group, mice were regularly followed after TC-1 implantation without specific treatment. For the imiquimod group, each mouse received topical imiquimod (50 mg/mouse) at the site of the tumor every two days (begun at d6 for a total of 6 treatments). For CRT/E7 alone group, each mouse was vaccinated with pcDNA3-CRT/E7 2 μg via gene gun at d9, d13, and d17. For the combined treatment group, each mouse received the same treatment schedule as for each monotherapy alone group. Tumors were harvested at d24 for flow cytometric analysis. Tumor cells were either stained with PE-conjugated anti-CD4 (L3T4) and FITC-conjugated anti-CD25 (PC61) mAbs for quantification of regulatory T cells, or stained with stained with PE-conjugated anti-CD11b (M1/70) and FITC-conjugated anti-Gr-1 (RB6-8C5) mAbs for quantification of MDSC.

For characterization of E7-specific CD8^+ ^T cells in tumor microenvironment, C57BL/6 mice were divided into four groups (untreated, imiquimod alone, CRT/E7 alone, and combined group). All mice were inoculated with TC-1 cells (5 × 10^4^/mouse) s.c. over right leg at d0. For the untreated group, mice were regularly followed after TC-1 implantation without specific treatment. For the imiquimod group, each mouse received topical imiquimod (50 mg/mouse) at the site of the tumor every two days (begun at d6 for a total of 6 treatments). For CRT/E7 alone group, each mouse was vaccinated with pcDNA3-CRT/E7 2 μg via gene gun at d9, d13, and d17. For the combined treatment group, each mouse received the same treatment schedule as for each monotherapy alone group. Tumors were harvested at d24 for immune response analysis. Before intracellular cytokine staining, pooled tumors from each treatment group were separately incubated for 16 h with the H2-D^b^-restricted CTL epitope (aa 49-57) (1.0 μmol/L). Cells were then harvested and stained for CD8 and IFN-γ using previously described standard protocols [19]. Samples were analyzed on a FACSCalibur flow cytometer using CellQuest software. All of the analyses shown were carried out on a gated lymphocyte population.

As for evaluation of change of various immune effectors in tumor microenvironment after imiquimod and CRT/E7 treatment, mice were divided into two groups (5/group: vehicle treated or imiquimod treated), and were implanted with TC-1 (5 × 10^4^/mouse) at d0. Each mouse received topical vehicle cream or imiquimod (50 mg/mouse) every two days initiated at d6 for a total of 6 times. Tumors were harvested 6 hours after last treatment. Tumor cells were then made into single cell suspension, washed once in FACScan buffer, and stained with surface markers for various innate and adaptive effectors including PE-conjugated anti-CD4 (L3T4), PE-conjugated anti-CD8 (53-6.7), FITC-conjugated anti-GR-1 (RB6-8C5), PE-conjugated anti-CD19 (1D3), PE-conjugated anti-NK1.1 (PK136), and PE-Cy5-conjugated anti-F4/80 (BM8). Cells were subjected to flow cytometry analysis gated on lymphocyte population.

### In vivo evaluation of the antitumor effects generated by combined treatment

In order to evaluate monotherapy alone or combined treatment for the inhibition of TC-1 tumor growth, we designed a two-step experiment. For the first-step experiment, we evaluated the anti-tumor effects of imiquimod alone therapy. Mice were divided into 2 groups (4/group, untreated or imiquimod treated), which were implanted with TC-1 (5 × 10^4^/mouse) subcutaneously at d0. Mice were then left untreated or treated with imiquimod (50 mg/mouse) at the site of the tumor every two days initiated at d6 for a total of 6 treatments. Mice were euthanized at d21 and tumors were harvested and photographed for tumor size comparison. After having proved that imiquimod was effective as a single agent, we proceeded with the second-step experiment. Mice were divided into four groups (5/group: untreated, imiquimod alone, CRT/E7 alone, and combined group). All mice were inoculated with TC-1 cells (5 × 10^4^/mouse) s.c. over right leg at d0. For the untreated group, mice were regularly followed for tumor growth after TC-1 implantation without specific treatment. For the imiquimod group, each mouse received topical imiquimod (50 mg/mouse) at the site of the tumor every two days (began at d6 for a total of 6 treatments). For CRT/E7 alone group, each mouse was vaccinated with pcDNA3-CRT/E7 2 μg via gene gun at d9, d13, and d17. For the combined treatment group, each mouse received the same treatment schedule as for each monotherapy alone group. Tumor sizes in the mice were monitored and measured every 3-4 days using digital calipers.

### In vivo antibody depletion experiments

In order to identify the subset of lymphocytes that were important for anti-tumor effects of combined treatments of imiquimod and pcDNA3-CRT/E7 DNA vaccine, mice were divided into 4 groups (combined treatments without depletion, combined treatments with depletion of neutrophils, combined treatments with depletion of NK1.1+ cells, and combined treatments with depletion of macrophages) using methods similar to what we have described previously [17]. All mice were implanted with TC-1 (5 × 10^4^/mouse) at d0, and were then treated with imiquimod (at d2, d4, d6, d8, d10, and d12) and pcDNA-CRT/E7 2 μg (d5 and d12). Mice were either undepleted or depleted of macrophages (using clodronate liposomes), NK1.1+ cells (using anti-mouse NK1.1 mAbs, PK136), or neutrophils (using anti-mouse Gr-1 mAbs, RB6-8C5), one day before and 3 days after TC-1 implantation and thereafter once per week until the end of follow-up.

### Statistical analysis

All data expressed as mean ± standard deviation (S.D.) are representative of at least two different experiments. Comparisons between individual data points for tumor sizes were made using a Student's *t*-test or repeated measure ANOVA (analysis of variance) test. Differences in survival between experimental groups were analyzed using the log-rank test. Tumor sizes were calculated using the following equation: (tumor length×width×height)/2. Death of mouse was arbitrarily defined as tumor diameter greater than 2 cm.

## Results

### Treatment with CRT/E7 DNA in combination with imiquimod leads to decrease in the number of myeloid-derived suppressor cells but not CD4+CD25+ T cells in the tumor microenvironment of tumor-bearing mice

**Figure 1:**
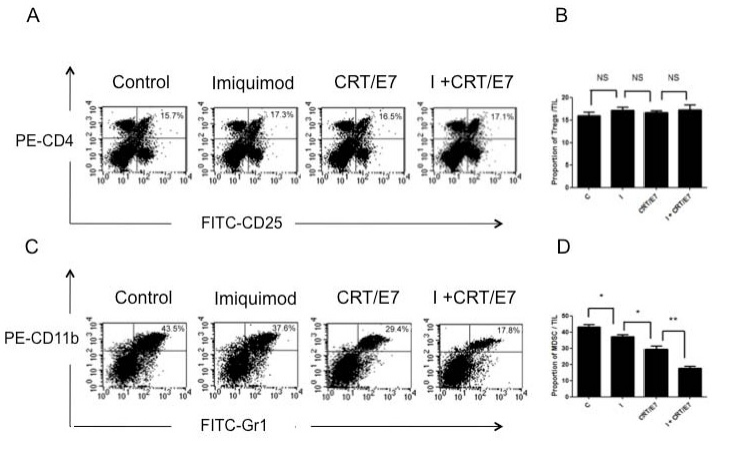
**Characterization of the T regulatory cells and myeloid-derived suppressor cells (MDSC) in the tumor microenvironment following vaccination with CRT/E7 DNA with or without imiquimod in tumor-bearing mice**. C57BL/6 mice were divided into four groups (5/group: untreated, imiquimod alone, CRT/E7 alone, and combined group). All mice were inoculated with TC-1 cells (5 × 10^4^/mouse) s.c. over right leg at d0. For the untreated group, mice were regularly followed after TC-1 implantation without specific treatment. For the imiquimod group, each mouse received topical imiquimod (50 mg/mouse) every two days (began at d6 for a total of 6 treatments). For CRT/E7 alone group, each mouse was vaccinated with 2 μg of pcDNA3-CRT/E7 DNA via gene gun at d9, d13, and d17. For the combined treatment group, each mouse received the same treatment schedule as for each monotherapy alone group. Tumors were harvested at d24 for flow cytometric analysis. Tumor cells were either stained with PE-conjugated anti-CD4 (L3T4) and FITC-conjugated anti-CD25 (PC61) mAbs **(A & B) **or with PE-conjugated anti-CD11b (M1/70) and FITC-conjugated anti-Gr-1 (RB6-8C5) mAbs **(C & D)**. Plots were gated on lymphocyte population. **A) **Representative flow cytometry data demonstrating the percentage of CD4+CD25+ cells. **B) **Bar graph representing the percentage of Tregs among the tumor-infiltrating lymphocytes. **C) **Representative flow cytometry data demonstrating the percentage of CD11b+Gr-1+ cells. **D) **Bar graph representing the percentage of Tregs among the tumor-infiltrating lymphocytes. C denotes untreated group, and I denotes imiquimod treated group. Representative data from one of three independent experiments are shown. *, P < 0.05; **, P < 0.01.

In order to determine the number of CD4+CD25+ T cells (which are mainly comprised of T regulatory cells) and myeloid-derived suppressor cells in the tumor microenvironment of tumor-bearing mice treated with CRT/E7 DNA with imiquimod, we inoculated C57BL/6 mice with TC-1 cells on d0 and treated them with or without topical imiquimod (50 mg/mouse) every two days starting from d6 for a total of 6 treatments). Mice were then vaccinated with CRT/E7 DNA via gene gun on d9, d13, and d17. Untreated mice were used as controls. Tumors were harvested on d24 and characterized for the presence of T regulatory cells and myeloid-derived suppressor cells by flow cytometry analysis. As shown in Figure 1, mice treated with CRT/E7 DNA in combination with imiquimod demonstrated significantly lower numbers of myeloid-derived suppressor cells compared to the other treated mice. In comparison, there was no significant difference in the number of CD4+CD25+ T cells in mice treated with CRT/E7 DNA in combination with imiquimod compared to any of the other groups. Thus, our data indicate that treatment with imiquimod in combination with DNA vaccination can reduce the number of myeloid-derived suppressor cells in the tumor microenvironment of treated mice.

### Treatment with CRT/E7 DNA in combination with imiquimod leads to an enhancement in the E7-specific CD8+ T cell immune responses in tumor-bearing mice

**Figure 2:**
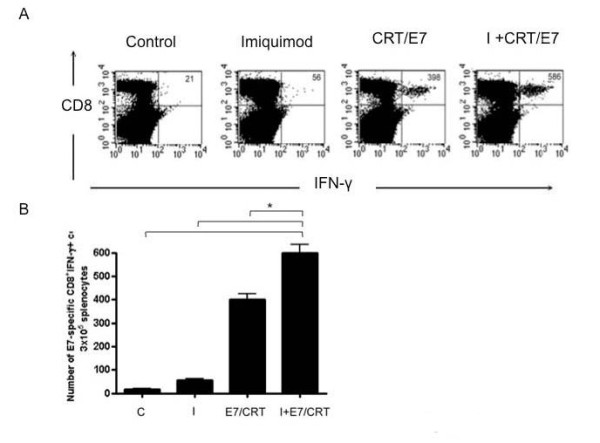
**Characterization of the E7-specific CD8+ T cell immune responses following vaccination with CRT/E7 DNA with or without imiquimod in tumor-bearing mice**. C57BL/6 mice were divided into four groups (5/group: untreated, imiquimod alone, CRT/E7 alone, and combined group). All mice were inoculated with TC-1 cells (1 × 10^4^/mouse) s.c. over right leg at d0. Treatment schedules were the same as in Figure 1. Splenocytes from tumor-bearing mice were harvested at d24 for immune response analysis, stimulated with E7 epitope (aa49-57), and stained for both CD8 and intracellular interferon-γ (IFN-γ). **A) **Representative flow cytometry data demonstrating the percentage of CD8^+^IFN-γ^+ ^T cells. The numbers of CD8^+ ^IFN-γ^+ ^double positive T cells are indicated in the upper right hand corner of each representative dot plot. **B) **Bar graph representing the number of E7-specific CD8+ T cells/3 × 10^5 ^splenocytes (*, p < 0.05). Representative data from one of three independent experiments are shown.

In order to determine the E7-specific immune responses in tumor-bearing mice vaccinated with CRT/E7 DNA with imiquimod, we inoculated C57BL/6 mice with TC-1 cells on d0 and treated them with CRT/E7 DNA vaccine and/or imiquimod as described in Figure 1. Splenocytes from tumor-bearing mice were harvested at d24 and characterized for the E7-specific CD8+ T cell immune responses by intracellular cytokine staining followed by flow cytometry analysis. As shown in Figure 2, mice treated with CRT/E7 DNA in combination with topical application of imiquimod demonstrated significantly higher numbers of E7-specific CD8+ T cells compared to mice treated with CRT/E7 alone. Thus, our data indicate that treatment with imiquimod in combination with DNA vaccination can significantly enhance the E7-specific CD8+ T cell immune responses in treated mice.

### Treatment with CRT/E7 DNA in combination with imiquimod leads to significantly improved antitumor effects and prolonged survival in treated mice

**Figure 3:**
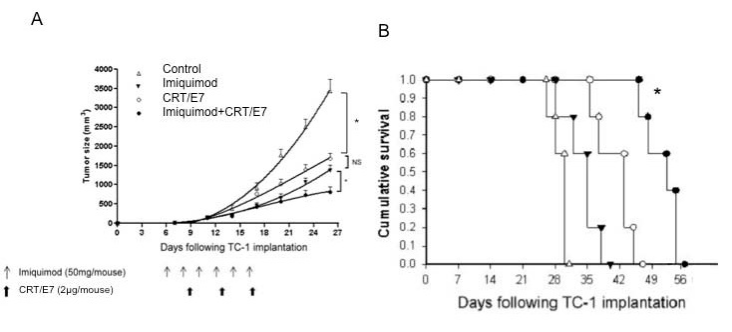
**Characterization of the therapeutic antitumor effects generated by treatment with E7-CRT in combination with imiquimod in tumor-bearing mice**. C57BL/6 mice (5/group: untreated, imiquimod alone, CRT/E7 alone, and combined group) were implanted with TC-1 (5 × 10^4^/mouse) at d0. Treatment schedules were the same as described in Figure 1. Tumor growth was monitored over time. **A) **Line graph depicting the tumor sizes in tumor-bearing mice treated with the different regimens. (*, p < .05) **B) **Kaplan-Meier survival curve of the mice treated with the various regimens (*, p < 0.05, compared between CRT/E7 alone and the combined group). Representative data from one of three independent experiments are shown.

In order to determine the therapeutic antitumor effects generated in tumor-bearing mice vaccinated with CRT/E7 DNA with imiquimod, we inoculated C57BL/6 mice with TC-1 cells on d0 and treated them with CRT/E7 DNA vaccine and/or topical application of imiquimod as described in Figure 1. Tumor growth was monitored over time. As shown in Figure 3A, mice treated with CRT/E7 DNA in combination with imiquimod demonstrated significantly lower tumor size over time compared to mice treated with CRT/E7 alone. Furthermore, mice treated with CRT/E7 alone or imiquimod alone demonstrated significantly lower tumor volumes compared to the mice without treatment (control). In addition, mice treated with CRT/E7 DNA in combination with topical application of imiquimod demonstrated significantly prolonged survival compared to mice treated with CRT/E7 alone (Figure 3B). Thus, our data indicate that topical treatment with imiquimod in combination with therapeutic HPV DNA vaccination can enhance the therapeutic antitumor effects against TC-1 tumors in treated mice.

### Treatment with imiquimod leads to increased number of NK1.1+ cells and F4/80+ cells in the tumor microenvironment

**Figure 4:**
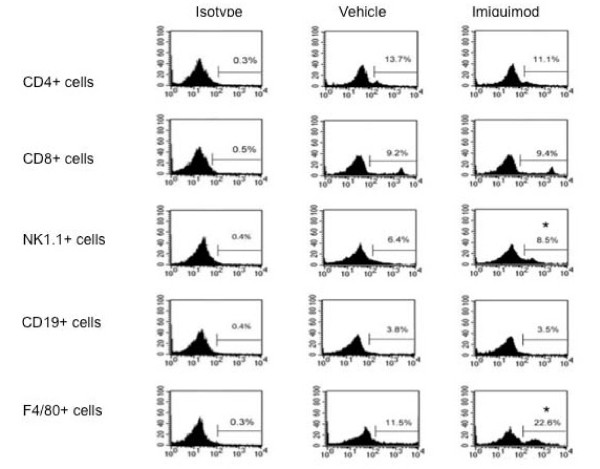
**Characterization of the levels of various immune cells in the tumor microenvironment following treatment with imiquimod**. C57BL/6 mice were divided into two groups (5/group: vehicle treated or imiquimod treated) and were implanted with TC-1 (5 × 10^4^/mouse) at d0. Each mouse received topical vehicle cream or imiquimod (50 mg/mouse) every two days initiated at d6 for a total of 6 times. Tumors were harvested 6 hours after last imiquimod treatment. Tumor cells were then made into single cell suspension, washed once in FACScan buffer, and stained with surface markers for various innate and adaptive effectors including PE-conjugated anti-CD4 (L3T4), PE-conjugated anti-CD8 (53-6.7), FITC-conjugated anti-GR-1 (RB6-8C5), PE-conjugated anti-CD19 (1D3), PE-conjugated anti-NK1.1 (PK136), and PE-Cy5-conjugated anti-F4/80 (BM8). Cells were subjected to flow cytometric analysis gated on lymphocyte population (*, p < 0.05, imiquimod versus vehicle). Representative data from one of three independent experiments are shown.

In order to determine the key immune cells in the tumor microenvironment that were upregulated following imiquimod treatment, C57BL/6 mice were implanted with TC-1 (5 × 10^4^/mouse) at d0 and divided into two groups (5/group: vehicle treated or imiquimod treated). Each mouse received topical vehicle cream or imiquimod every two days initiated at d6 for a total of 6 times. Tumors were harvested 6 hours after last imiquimod treatment. Tumor cells were then made into single cell suspension and analyzed for the presence of various immune cell markers by flow cytometry analysis. As shown in Figure 4, mice treated with topical application of imiquimod demonstrated significantly higher number of NK1.1+ cells, which includes both NK cells as well as NKT cells, and F4/80+ inflammatory cells in the tumor microenvironment compared to mice treated with the vehicle. Thus, our data indicate that imiquimod treatment leads to the upregulation of NK1.1+ cells and F4/80+ inflammatory cells in the tumor microenvironment.

### Macrophages and NK1.1+ cells play an important role in the antitumor effects mediated by treatment with CRT/E7 DNA in combination with imiquimod

**Figure 5:**
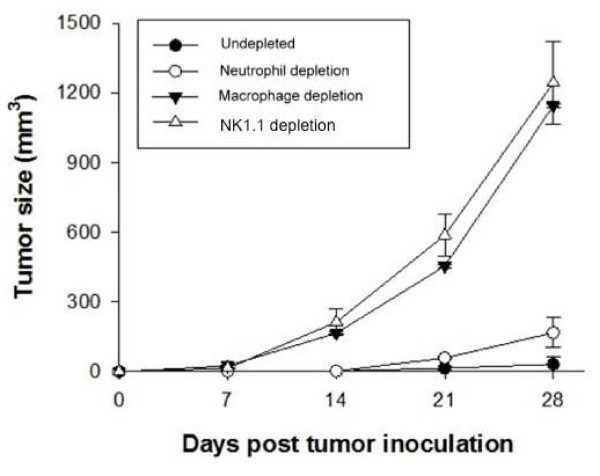
**In vivo depletion experiment**. C57BL/6 mice were divided into 4 groups (5/group). All mice were implanted with TC-1 (5 × 10^4^/mouse) at d0, and were then treated with imiquimod (at d2, d4, d6, d8, d10, and d12) and pcDNA-CRT/E7 2 μg (d5 and d12). Mice were either depleted of macrophages (using clodronate liposomes), NK1.1+ cells (using anti-mouse NK 1.1 mAbs, PK136), or neutrophils (using anti-mouse Gr-1 mAbs, RB6-8C5), one day before and 3 days after TC-1 implantation and thereafter once per week until the end of follow-up. Undepleted mice were used as controls. Line graph depicting the tumor sizes in tumor-bearing mice treated with the different regimens. Representative data from one of three independent experiments are shown.

In order to determine the immune cells that play an important role in the antitumor effects mediated by CRT/E7 DNA vaccination followed by imiquimod treatment, we performed an in vivo depletion experiment. C57BL/6 mice were divided into 4 groups (5/group). All mice were implanted subcutaneously with TC-1 at d0, and were then treated with imiquimod and CRT/E7 DNA vaccine as described. Mice were either depleted of macrophages (using clodronate liposomes), NK1.1+ cells (using anti-mouse NK 1.1 mAbs, PK136), or neutrophils (using anti-mouse Gr-1 mAbs, RB6-8C5), one day before and 3 days after TC-1 implantation and thereafter once per week until the end of follow-up. Undepleted mice were used as controls. As shown in Figure 5, treated mice depleted of macrophages or NK1.1+ cells demonstrated significant increase in tumor growth compared to undepleted mice or mice depleted of neutrophils. Thus, our data indicate that macrophages and NK1.1+ cells play an important role in the antitumor effects mediated by treatment with CRT/E7 DNA in combination with imiquimod.

## Discussion

In the current study, we observed that treatment with CRT/E7 DNA in combination with imiquimod leads to decrease in the number of myeloid-derived suppressor cells but not CD4+CD25+ T cells in the tumor microenvironment of tumor-bearing mice. Treatment with CRT/E7 DNA in combination with imiquimod was also found to lead to an enhancement in the E7-specific CD8+ T cell immune responses and improved antitumor effects and prolonged survival in treated mice. Macrophages and **NK1.1**+ cells were found to play an important role in the antitumor effects mediated by treatment with CRT/E7 DNA in combination with imiquimod. Our data is quite consistent with the recent clinical trials using therapeutic HPV vaccines in conjunction with imiquimod in patients with vulvar intraepithelial neoplasia. In that study, treatment with imiquimod and vaccination led to clinical responses in some of the patients, with a significant increase local infiltration of CD8+ and CD4+ T cells in the lesions of these patients [20].

In our study, we have observed that depletion of macrophages in TC-1 tumor-bearing mice treated with imiquimod and CRT/E7 DNA vaccines led to the loss of antitumor effect, suggesting the importance of macrophages in the antitumor effect generated by imiquimod in combination with the DNA vaccine (Figure 5). Tumor-bearing mice treated with imiquimod also showed a significant increase in the number of F4/80+ macrophages in the tumor microenvironment (Figure 4). Taken together, these data suggest that the combination of CRT/E7 DNA vaccination with imiquimod treatment may result in an increase in M1 macrophages in the tumor, which can contribute to the antitumor effect (for reviews see [21,22]) It has been shown that TC-1 tumors contain significant number of M2 macrophages, that lead to suppression of antitumor T-cell response, thus facilitating tumor growth [23]. Thus, treatment of TC-1 tumors with imiquimod and the CRT/E7 DNA vaccine may result in a switch from immunosuppressive M2 macrophage phenotype to the inflammatory M1 phenotype.

We showed that treatment with imiquimod leads to increased number of NK1.1+ cells and F4/80+ cells in the tumor microenvironment (Figure 4). Furthermore, our antibody depletion experiments indicated that NK1.1+ cells are essential for the observed antitumor effects (Figure 5). NK cells have been shown to express TLR7 and imiquimod-mediated activation of NK cells through TLR7 may result in IFN-γ production [24]. Thus, it is likely that not only can NK cells directly lead to cytotoxic effects, but NK cells can also lead to increased expression of IFN-γ which may lead to upregulation of MHC class I, which may render tumor cells more susceptible to killing by CD8+ T cells.

We observed that treatment with CRT/E7 DNA in combination with imiquimod leads to a reduction in the number of myeloid-derived suppressor cells (MDSCs) in the tumor microenvironment of tumor-bearing mice (Figure 1). It has been shown that myeloid-derived suppressor cells play an important immunosuppressive role in the tumor microenvironment (for reviews, see [25-27]). The reduction in MDSCs may potentially lead to improved antigen-specific CD8+ T cell immune responses, resulting in better tumor killing.

The employment of imiquimod for tumor treatment has significant potential for clinical translation. Currently there is one ongoing phase I trial to assess the immunogenicity, safety, tolerability and efficacy of pNGVL4a-Sig/E7(detox)/HSP70 DNA vaccine [28] followed by a booster vaccination with a recombinant vaccinia expressing HPV-16/18 E6 and E7 (TA-HPV) [29] in combination with locally applied TLR7 agonist, imiquimod in patients with HPV-16+ high grade cervical intraepithelial neoplasia (CIN3) http://www.clinicaltrial.gov/ct2/show/NCT00788164. The application of imiquimod may generate local inflammation in the local lesional microenvironment, thus allowing the localized immune effector cells to access lesional epithelium in patients with CIN lesions, and function in tumor destruction in the local microenvironment.

In summary, our study demonstrates that the combination of therapeutic HPV DNA vaccination with topical treatment with the TLR7 agonist, imiquimod enhances the antitumor immunity induced by DNA vaccination. Our study serves as an important foundation for future clinical translation.

## Competing interests

The authors declare that they have no competing interests.

## Authors' contributions

CMC was involved in the execution of the project. AM was involved in the interpretation of the data and writing the manuscript. CFH and TCW provided overall supervision and guidance for the project. All authors read and approved the manuscript.
